# The mismatch between experimental and computational fluid dynamics analyses for magnetic surface microrollers

**DOI:** 10.1038/s41598-023-37332-5

**Published:** 2023-06-23

**Authors:** Ugur Bozuyuk, Hakancan Ozturk, Metin Sitti

**Affiliations:** 1 Physical Intelligence Department, Max Planck Institute for Intelligent Systems, 70569 Stuttgart, Germany; 2 Institute for Biomedical Engineering, ETH Zurich, 8092 Zurich, Switzerland; 3 School of Medicine and School of Engineering, Koç University, Istanbul, 34450 Turkey

**Keywords:** Applied physics, Fluid dynamics, Biomedical engineering, Chemical engineering, Mechanical engineering

## Abstract

Magnetically actuated Janus surface microrollers are promising microrobotic platform with numerous potential biomedical engineering applications. While the locomotion models based on a "rotating sphere on a nearby wall" can be adapted to surface microrollers, real-world dynamics may differ from the proposed theories/simulations. In this study, we examine the locomotion efficiency of surface microrollers with diameters of 5, 10, 25, and 50 µm and demonstrate that computational fluid dynamics simulations cannot accurately capture locomotion characteristics for different sizes of microrollers. Specifically, we observe a significant mismatch between lift forces predicted by simulations and opposite balancing forces, particularly for smaller microrollers. We propose the existence of an unaccounted force component in the direction of lift, which is not included in the computational fluid dynamics simulations. Overall, our findings provide a deeper understanding of the physical mechanisms underlying surface microroller locomotion and have important implications for future applications in biomedical engineering.

## Introduction

Magnetic surface microrollers have shown great potential for various biomedical applications, such as navigation in blood flow for cargo delivery and potential lab-on-a-chip applications^1–14^. The translational motion of the microrollers is achieved by the rotation of the particle body on a nearby wall, by applying external uniform rotating fields^15–18^. The locomotion direction can also be precisely controlled by changing the orientation of the rotational field^1–4^. To better understand the locomotion characteristics of surface microrollers and improve their practical applications, proposed theories of "rotating sphere on a nearby wall" can be employed^15,16,18^. Such theories provide a valuable framework for studying the behavior of these microrollers and optimizing their performance.

**Figure 1:**
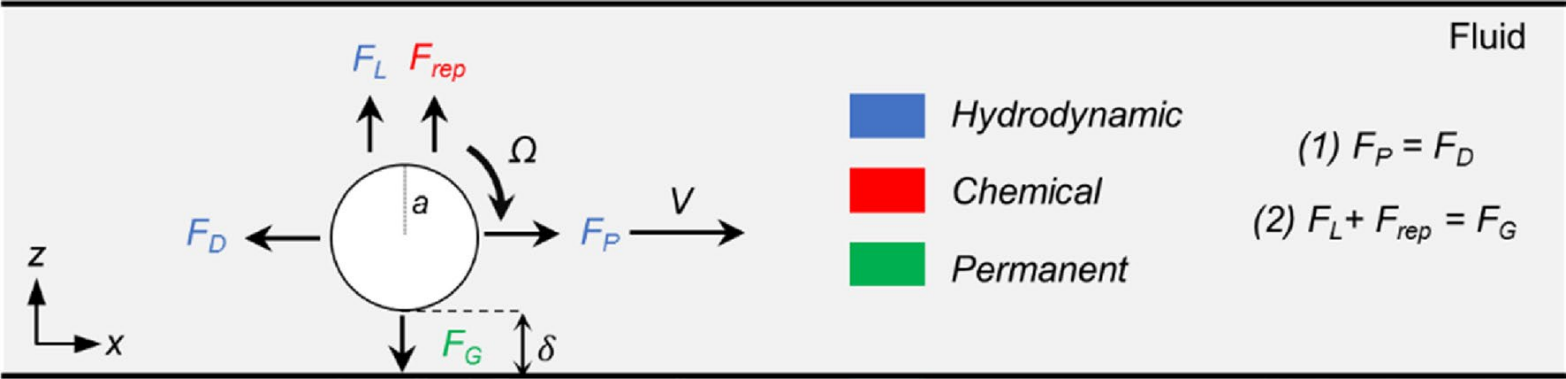
The basic force balance on a microroller near a wall. A microroller, rotating with an angular velocity of *Ω*, with a distance of $\delta$ from the nearby wall, creates a propulsion force, *F*_*P*_, balanced out the drag force, *F*_*D*_, on the *x*-axis. The lift force, *F*_*L*_, and repulsion force, *F*_*rep*_, are balanced by the gravitational over-buoyancy force, *F*_*G*_. The origin of the forces was marked with the color code.

Asymptotic solutions of the Stokes equations^15^ are proposed to explain the locomotion of the rotating sphere on a nearby wall in the low Reynolds number regime, which also has been used for surface-rolling microrobots^1–3,6,19–21^. The theory explains the force balance on the *x*-axis, that the sphere's rotation creates a propulsion force, *F*_*P*_, which is balanced out by a drag force, *F*_*D*_, due to the translational motion of the sphere (Fig. 1) ^15^. On the other hand, the extent of these forces also depends on the lubrication distance, $\delta$, which is the result of the force balance on the *z*-axis (Fig. 1). Unlike the forces in the *x*-axis, there are forces with non-hydrodynamic origins, such as *F*_*rep*_ and *F*_*G,*_ in the *z*-axis (Fig. 1).* F*_*rep*_ is the chemical repulsion force between the sphere and the wall^22^, and *F*_*G*_ is gravitational over buoyancy, a permanent non-contact force depending on the material properties^23^. On the other hand, the lift force, *F*_*L*_, has a hydrodynamic origin, which explains the hydrodynamic repulsion of the sphere due to flows created by the nearby wall (Fig. 1) ^24–26^. To capture the forces with a hydrodynamic origin, we developed a computational fluid dynamics (CFD) simulation in three dimensions for the rotating and translating sphere case^2^ that converged the same results from Goldman et al. ^2,15^. Therefore, we can study the nature of these forces for different environments and sizes for different conditions.

In this work, we demonstrated how experimental results were mismatched with the CFD simulation results for surface microrollers. We performed basic experimental analysis on the spherical microrollers of different diameters, 5, 10, 25, and 50 µm. We analyzed their translational speeds (*V*) in a static and semi-infinite environment for increasing rotational speeds (*Ω*). Our investigation revealed a non-linear relationship between microroller size and locomotion efficiency, defined as the microroller's ability to convert rotational motion into translational motion. The microroller with a diameter of 50 µm demonstrated the highest efficiency among the different sizes tested, while the 10 µm microroller was found to be the least efficient. The overall ranking of the microrollers from most to least efficient was as follows: 50 µm > 5 µm > 25 µm > 10 µm. To understand this relationship, we conducted computational fluid dynamics (CFD) analyses. However, the analyses failed to explain the irregular relationship observed in the experimental results. In fact, the CFD analyses significantly overestimated the forces involved, with *F*_*G*_ dominating over *F*_*L*_ and *F*_*rep*_. This discrepancy indicated that the force balance on the *z-*axis was not satisfied. Overall, the main aim of this study is to analyze this discrepancy and discuss the reasons behind it.

## Results

### The basic speed analyses of microrollers with different diameters

**Figure 2:**
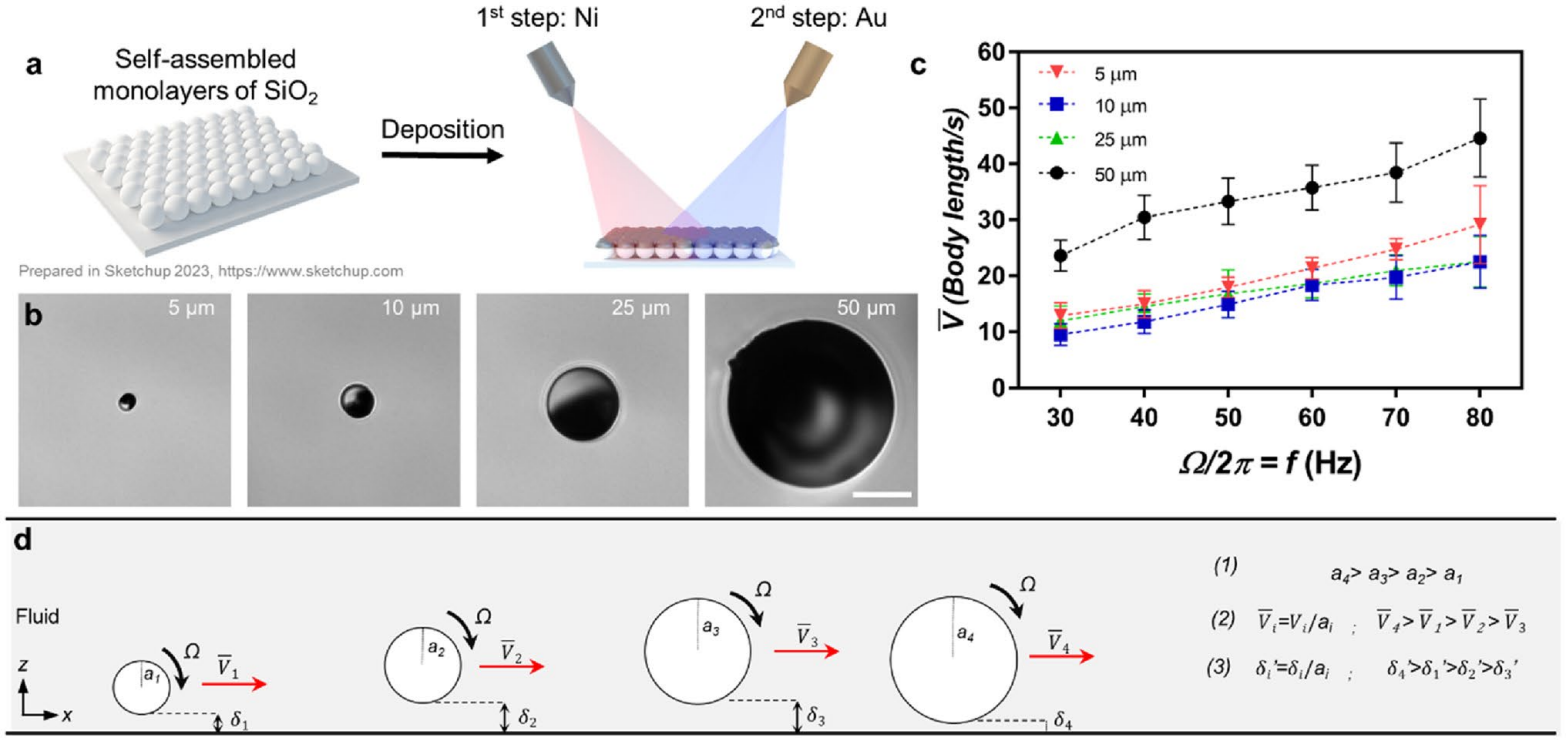
Basic speed analyses of magnetic surface microrollers with different sizes. (**a**) The schematic representation of the fabrication process to fabricate surface microrollers. Monolayers of SiO_2_ microparticles with different diameters were prepared on glass substrates. Then magnetic material (Ni) and passivating layer (Au) were sputter-coated on the particles. After sputter coating, the magnetic film was programmed in the out-of-plane direction by applying a 1.7 T uniform magnetic field while the Janus particles were still on the glass substrate. The schematic was prepared with Sketchup 2023 software, https://www.sketchup.com. (**b**) Light microscopy images from surface microrollers with different sizes ≈ 5, 10, 25, and 50 μm diameters were used in the study. The scale bar is 20 μm. (**c**) The average relative translational speeds of the microrollers depend on increasing rotation frequencies. The most efficient microroller depending on the body length per second was 50 μm and then 5 μm. The least efficient one was 10 μm. (**d**) A summary of the experimental results. The most efficient microroller was the biggest one, but there was a non-monotonic relation between diameters and speeds, which has to do with their relative lubrication distance.

We characterized the speeds of magnetic surface microrollers for different sizes (Fig. 2). The fabrication of surface microrollers is summarized in Fig. 2a. The silica particles with different sizes were monolayered on a glass substrate, and then the particles were sputter-coated with Ni and Au. To obtain ≈5 μm microroller, 4 μm silica particles are sputter coated with 1000 nm Ni and 50 nm Au; the ≈10 μm microroller also has 1000 nm Ni and 50 nm Au on top of 10 μm particles (Fig. 2b). The ≈25 and ≈50 μm microrollers contain 1800 nm Ni and 50 nm Au on top of 25 and 50 μm silica particles, respectively (Fig. 2b). Then, we characterized their steady-state translational speeds for increasing rotational frequencies until* f* = 80 Hz since it was the highest actuation frequency for 50 μm (Figure S1); therefore, *f* = 80 Hz was selected for the highest limit for all groups for a concise comparison. The other microrollers did not also step-out until *f* = 80 Hz as their translational speed never decreased with increasing rotation frequency (Fig. 2c). The 50 μm microrollers performed best with the highest body length per second (Fig. 2c), and 5 μm microrollers had the second ranking. The performances of the 10 and 25 μm microrollers were close to each other; the 25 μm had slightly higher relative velocity (Fig. 2c). The relative velocity of the microrollers, their translational speed over their diameter, *V/2a*, was determined by the lubrication distance, ${\delta }^{^{\prime}}=\delta /a$. The microrollers would have the same relative velocity at a fixed ${\delta }^{^{\prime}}$ for a specific rotation frequency; however, ${\delta }^{^{\prime}}$ is deterministically reached for a specific frequency and microroller size depending on the forces on *z-*axis. Thus, the relative speed differences were due to different ${\delta }^{^{\prime}}$ values for different sizes; smaller ${\delta }^{^{\prime}}$ results in more efficient microroller locomotion. Therefore, we could say that the 50 μm microroller had the smallest ${\delta }^{^{\prime}}$, while 10 μm had the highest (Fig. 2d), and the overall ranking goes as ${{\delta {^{\prime}}}_{10 \mathrm{\mu m} }>{\delta {^{\prime}}}_{25 \mathrm{\mu m} }>{\delta {^{\prime}}}_{5 \mathrm{\mu m} }>{\delta {^{\prime}}}_{50 \mathrm{\mu m} }}$. The value of ${\delta }^{^{\prime}}$ depends on the force balance of the microroller on the *z-*axis (Fig. 1).

### The governing forces of microrollers at different sizes in the CFD environment

**Figure 3:**
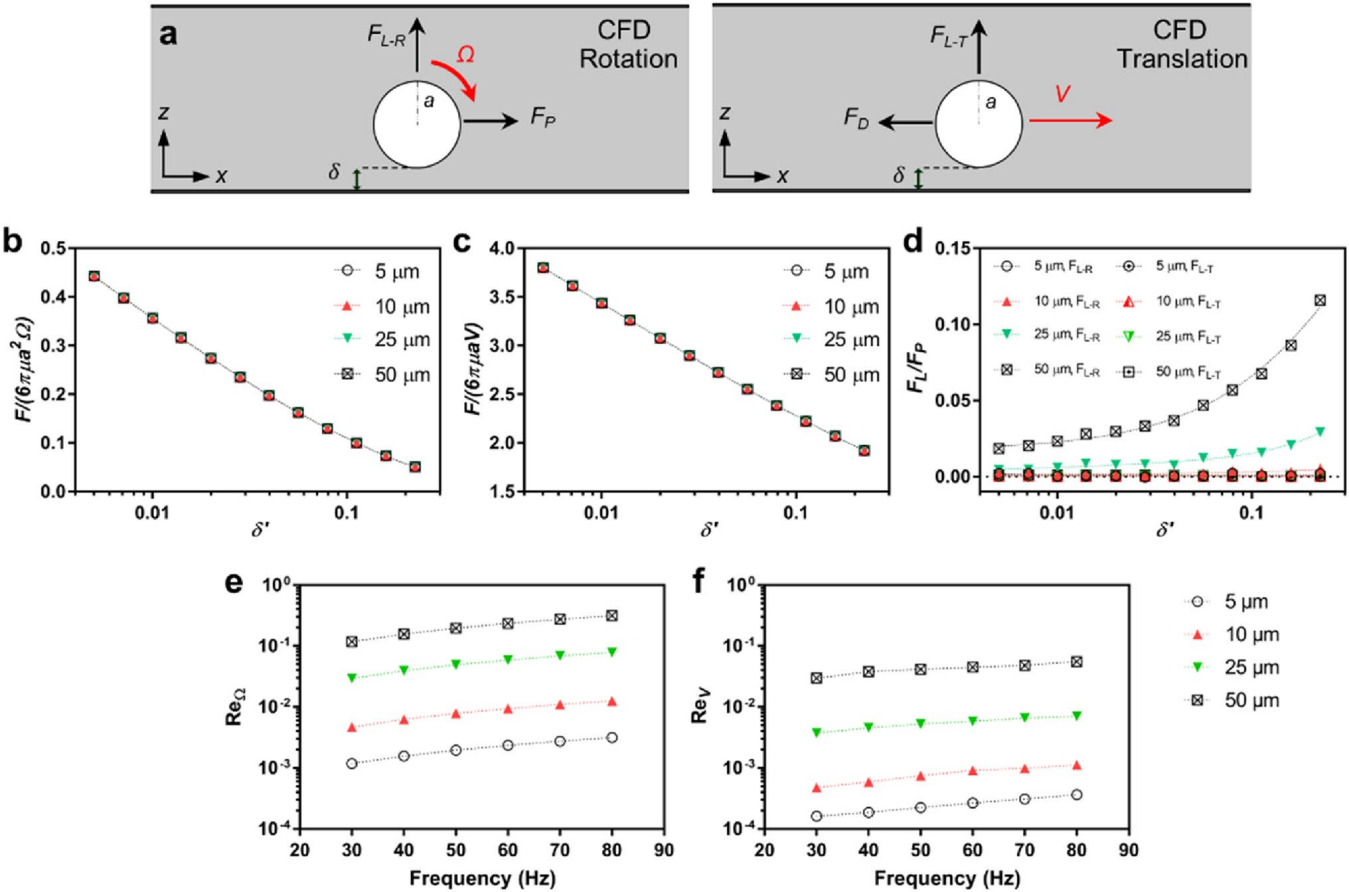
The force balance on a rotating sphere/microroller and CFD simulations. (**a**) The summary of CFD simulations. A sphere in 3 dimensions was either rotated or translated. Natural motion is combined motion, where rotation and translation occur simultaneously. The cases were analyzed in steady state conditions to investigate the governing forces. (**b,c**) The normalized force on a rotating and translating sphere for different sphere diameters depending on the lubrication distance. The results show that the normalized forces overlap for different diameters in both cases. (**d**) The relative lift forces on the rotating and translating sphere for both cases. The lift forces appear after a rotating sphere starting from the 25 μm diameter. The lift forces seem to be negligible for smaller microrollers. Also, the contribution of the translational lift force, *F*_*L-T*_ is negligible compared to the rotational lift force, *F*_*R-T*_. (**e,f**) Rotational (*Re*_*Ω*_) and translational (*Re*_*V*_) Reynolds numbers based on the experimental results in Fig. 2c.

We performed CFD simulations to capture the forces with hydrodynamic origins, such as *F*_*P*_*, F*_*D,*_ and *F*_*L,*_ for different microroller sizes. We performed CFD simulations in 3D with two different configurations (Fig. 3a), pure rotation and translation, where the natural motion of the microroller is a combination of the two. We can investigate the system by decomposing these two cases due to the linearity of motion and stress Eqs. ^15^. We quantified the forces acting on the microroller in both *x-* and *z-*axes. The forces on the *x-*axis in the pure rotation and translation case correspond to *F*_*P*_ and *F*_*D*_, respectively^2,15^. The forces in the *z-*axis give the *F*_*L*_, while the translation and rotation have different contributions to the total *F*_*L*_. We performed the simulations for all microroller sizes where the environments were adjusted to 40*a* × 40*a* × 40*a* to avoid any confinement effects^2^. We rotated all microrollers for *f* = 30, 50, 75 and 100 Hz and *V* = 30, 50, 75 and 100 body length per second for different $\delta {^{\prime}}$ values (Fig. 3b-d) to be able to derive the expressions for the governing forces. The results revealed that the relative *F*_*P*_ and *F*_*D*_ values overlapped for all sizes (Fig. 3b,c), implying that these two have no size scaling effect. Furthermore, we used a finer mesh for the simulations than the previous work^2^ that was validated from the work from Goldman et al. ^15^ to ensure more reliable results. Therefore, we obtained new expressions for *F*_*P*_ and *F*_*D*_ with modified correction factors (R^2^ > 0.99 for both expressions):

1 $${F}_{P}=6\pi \mu {a}^{2}\Omega \left(-0.1045ln({\delta }^{^{\prime}}\right)-0.1268)$$

2 $${F}_{D}=6\pi \mu aV\left(-0.4975ln({\delta }^{^{\prime}}\right)+1.1405)$$

On the other hand, the lift forces were negligible for the size below 25 μm, and very prominent for 50 μm (Fig. 3d). The other important finding is that the translational motion did not cause any *F*_*L*_, demonstrating the rotational force is the mere contributor to the lift force. We also derived an expression for *F*_*L*_ with the new correction factors using the result of 50 μm since CFD loses the sensitivity in *F*_*L*_ for smaller sizes (R^2^ > 0.99):

3 $${F}_{L}={\rho }_{f}{a}^{4}{\Omega }^{2}\left(-0.0382ln({\delta }^{^{\prime}}\right)+0.353)$$

We found out that *F*_*P*_ and *F*_*D*_ had no scaling effect, while *F*_*L*_ started appearing with bigger microroller sizes. When we quantified rotational (*Re*_Ω_) and translational (*Re*_*V*_) Reynolds numbers based on the experiments in Fig. 2c (Fig. 3e,f), we observed that the *Re*_Ω_ for 25 and 50 μm reaches around 0.1–0.4, indicating that inertial forces played some role on the locomotion of such microrollers, while the contribution of translational locomotion was still small. This also agreed well with the *F*_*L*_ quantification (Fig. 3d), since the nature of *F*_*L*_ is inertial^26^. Moreover, it is worth highlighting that the expressions for propulsion force (*F*_*P*_) and drag force (*F*_*D*_) put forth for the low Reynolds number regime^15^ remained valid even when some degree of inertia was introduced into the system for the range of parameters studied (Fig. 3b,c,e,f). To better understand the experimental behavior, we now turn our attention to the effects of other forces.

### Generic force relations between the governing forces

**Figure 4:**
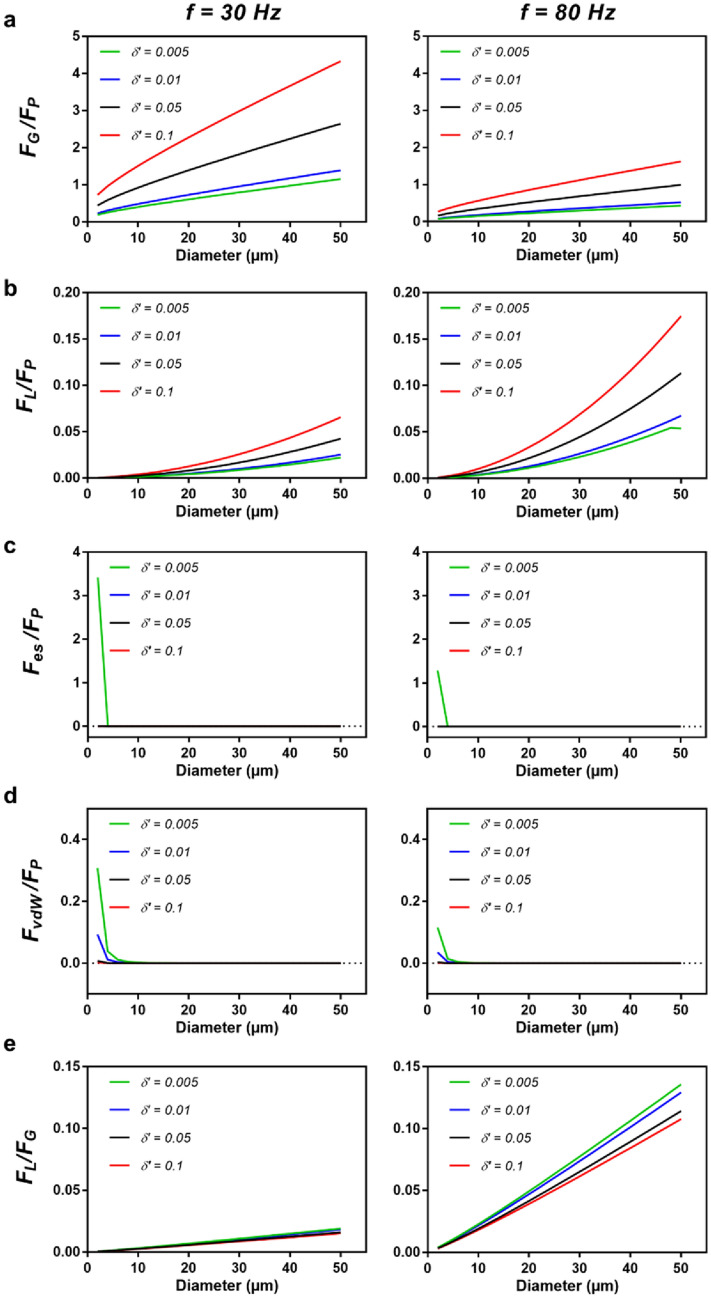
Generic force relations between different forces depending on microroller diameter for varying delta at *f* = 30 and 80 Hz. (**a**) The relation between gravitational force (*F*_*G*_) and propulsion force (*F*_*P*_) for 30 and 80 Hz for different ${\delta }^{^{\prime}}$. Gravitational forces start to become more prominent at greater microroller diameters. (**b**) The relation between the lift force (*F*_*L*_) and the propulsion force (*F*_*P*_). At smaller diameters, lift forces were less pronounced. At higher diameters, at ${\delta }^{^{\prime}}$=0.1, only 20% of the propulsion force was generated, unlike the gravitational forces. (**c**) The relation between electrostatic repulsion forces and propulsion forces. Electrostatic forces were only prominent when the microroller was very close to the wall, ${\delta }^{^{\prime}}$=0.005, and became invisible at higher microroller diameters and lubrication distances; therefore, it is assumed to be negligible. (**d**) The relation between van der Waals attraction forces and propulsion forces. The magnitude of the forces were negligible in the size regime of 5–50 μm; therefore, their effect is assumed to be negligible. (**e**) The relation between the lift force (*F*_*L*_) and the gravitational forces. The theoretical lift forces seem to be only around 10% of the gravitational forces calculated, while they must have been in balance.

As demonstrated in the previous section, the *F*_*L*_ only becomes noticeable in the CFD environment for microrollers with diameters greater than 25 μm, with the most significant effect observed for 50 μm microrollers. This suggests that larger microrollers may generate greater *F*_*L*_ and, therefore may have a higher ${\delta }^{^{\prime}}$. However, other forces in the force balance may counteract the effects of *F*_*L*_ and alter this relationship. In this section, we explored the relationship between forces acting in the *z*-axis for microrollers of various sizes and frequencies. This section investigates the relationship between the forces in the *z-*axis for different sizes and frequencies. We calculated the *F*_*G*_ using the experimentally calculated densities of the microrollers and interpolated the sizes between them. *F*_*P*_ and *F*_*L*_ were also calculated for all sizes using Eqs. (2) and (4) for *f* = 30 and 80 Hz for different ${\delta }^{^{\prime}}$. *F*_*rep*_ was approximated to be the difference between electrostatic repulsion forces (*F*_*es*_) and van der Waals attraction forces (*F*_*vdW*_) such that *F*_*rep*_ = *F*_*es*_ + *F*_*vdW*_ (Sup. Note 1) for the same ${\delta }^{^{\prime}}$ ^27^. The computed forces in the *z*-axis were normalized to the corresponding *F*_*P*_ values for a concise comparison between different sizes. The results are summarized in Fig. 4. In this section, we demonstrated the results for the diameter of the microroller vs the force relation *(e.g.*, *F*_*G*_*/F*_*P*_) for different ${\delta }^{^{\prime}}$. Since the experimental value of ${\delta }^{^{\prime}}$ is unknown and difficult to measure^28^, we presented broad range of ${\delta }^{^{\prime}}$. Also, it is known that ${\delta }^{^{\prime}}$ increases with increasing *f* ^2,28^; therefore, the higher ${\delta }^{^{\prime}}$ represents high relative *f* region and vice versa.

As expected, the gravitational forces increased with the microroller diameter (Fig. 4a). They were more dominant at *f* = 30 Hz due to less *F*_*P*_ generated and even went up to roughly four times than *F*_*P*_ for 50 μm at ${\delta }^{^{\prime}}=$ 0.1. The effect of *F*_*G*_ disappeared remarkably for smaller diameters, especially at small lubrication distances. *F*_*G*_ seemed less important at higher frequencies, such as *f* = 80 Hz (Fig. 4a). *F*_*L*_ was also not prominent for smaller diameters and frequencies (Fig. 4b). The magnitude of *F*_*L*_ is insignificant compared to *F*_*P*_ almost for all sizes, and it went a maximum of 20% of the *F*_*P*_ at 50 μm, ${\delta }^{^{\prime}}=$ 0.1 (Fig. 4b). One should note that the *y-*axis of the graphs in Fig. 4a,b are significantly different, implying that *F*_*G*_ is a more dominant force than *F*_*L*_. It was also revealed that the components of *F*_*rep*_ was only prevalent when the size is less than 3–4 μm and the microroller was very close to the wall (Fig. 4c,d). As *F*_*rep*_ is a surface-related chemical force, we expected it to become more significant as the surface-to-volume ratio increased. Due to the size range of our microrollers, we did not observe a significant contribution from *F*_*rep*_ and therefore did not include it in our further discussions.

As in our study, the forces on the *z-*axis must balance each other on steady-state locomotion; however, that was not the case for our system (Fig. 1, Fig. 4e). As a result, the magnitude *F*_*L*_ is much smaller than the *F*_*G*_ for all conditions (Fig. 4e), reaching a maximum of ≈10% for *f* = 80 Hz at higher microroller diameters. Since *F*_*G*_ is a permanent force and thus cannot change, the *F*_*L*_ generated by the microrollers seemed to be missing in CFD simulations. There is also a discrepancy in theoretical analysis, the division of* F*_*L*_ over *F*_*G*_ yields:

4 $$\frac{{F}_{L}}{{F}_{G}}=\frac{{\rho }_{f}a{\Omega }^{2}\left(-0.0382ln({\delta }^{^{\prime}}\right)+0.353)}{\frac{4}{3}\pi {(\rho }_{p-}{\rho }_{f})g }=1$$

When the diameter (*2a*) of the microroller increases at constant $\Omega$, the ${\updelta }^{\mathrm{^{\prime}}}$ also tries to increase itself to keep the equation in balance. However, this was not the case for experiments, and there was an irregular relation between size and the translational speed of the microrollers. Overall, it is evident that there is a missing force on the *z-*axis, which CFD analyses could not capture.

### The missing/balancing force on the *z*-axis

**Figure 5:**
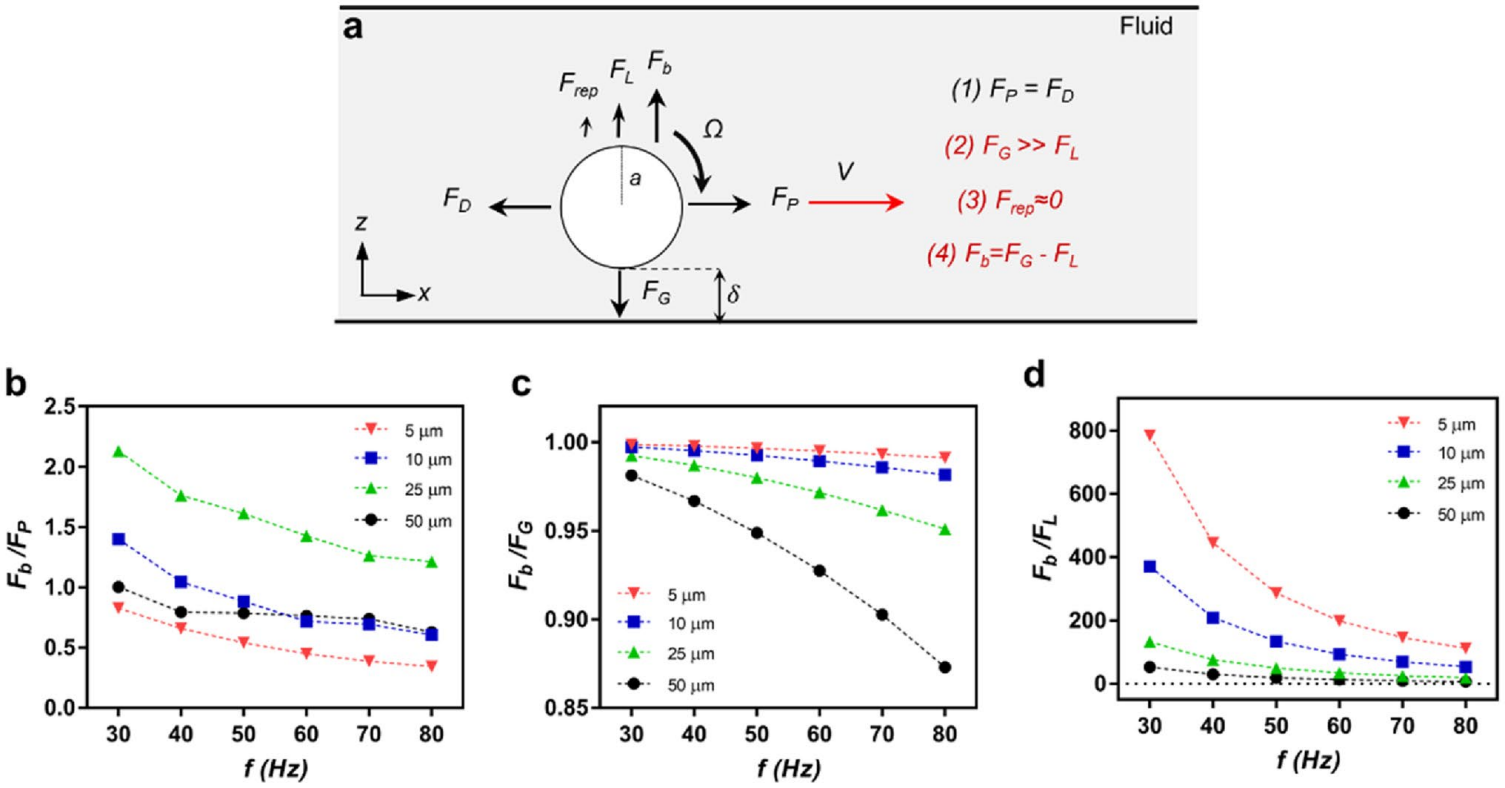
The modified force balance for our experimental system. (**a**) The proposed force balance, including the new balancing force, *F*_*b*_, to balance out the *F*_*G*_. (**b**) The relation between the balancing force (*F*_*b*_) and *F*_*P*_. (**c**) The relation between the balancing force (*F*_*b*_) and *F*_*G*_. The analyses revealed a significant force is missing for 5 μm, as big as *F*_*G*_ itself. (**d**) The relation between the balancing force (*F*_*b*_) and *F*_*L*_.

We propose a new force balance for our system, demonstrated in Fig. 5a. A new force must fill the missing force, *F*_*b*_, to balance out the *z-*axis. We compared *F*_*b*_ with *F*_*P*_ for different rotation frequencies to get an initial glance. The analyses revealed that *F*_*b*_ is a significant force, having similar and even greater values than* F*_*P*_ (Fig. 5b). Then we compared the *F*_*b*_ with *F*_*G*_; the analysis demonstrated that the missing/balancing force, *F*_*b*_, is so significant that it is almost entirely missing for 5 μm microroller (Fig. 5c). The *F*_*b*_ followed a regular pattern depending on the size of the microroller when compared to *F*_*G*_, meaning that it was the biggest for 5 μm and smallest for 50 μm, even though the ratios were significantly high for all groups (Fig. 5c). Compared to the *F*_*L*_, *F*_*b*_ is much greater than *F*_*L*_, showing that the magnitudes of *F*_*L*_ are very small in CFD (Fig. 5d).

## Discussions

In this work, we found out that the forces on the *z*-axis were not balancing each other out in the theoretical analyses. The missing force was in the direction of the lift force, *F*_*L*_, which had a similar order with *F*_*G*_. The possible reasons why CFD could not fully capture the experimental characteristics are as follows:

1. The accuracy of CFD simulations can be limited by assumptions such as no-slip boundary conditions^29,30^, which may not be optimal for mesoscale cases, as found here^30^. This approximation can lead to under-/overestimation of forces and the omission of important features such as atomic and surface forces, wetting, and physio-chemical parameters^29,30^. Therefore, for example, As a result, the quantified lift force in simulations may not reflect the actual behavior of microrollers. For example, previous experimental work has shown that 10 μm microroller lifts itself with increasing *f* , in other words, ${\delta }^{^{\prime}}$ has increased with increasing* f*, at low Reynolds number regime where lift forces do not exist in CFD simulations^28^.
2. Although CFD simulations are useful for predicting the behavior of microrollers, they may oversimplify the geometry of the objects and overlook important experimental properties. For example, the surface roughness of the nearby wall and the Janus structure of microrollers are not always considered in these simulations^15,29^. For example, recent study by Rinehart et al. has demonstrated that a significant lift force arises from a rotating Janus particle in the Stokes regime due to slip inhomogeneity in the particle body^31^. Other than roughness or body inhomogeneity, slight shape anisotropies in the body of the microrollers due to the thin film deposition may be a contributing factor. To study the effect of anisotropy, we briefly modeled anisotropic microrollers and observed that the contribution of experimental anisotropy is almost negligible (Sup. Note 2). Also, wet friction caused by the surrounding fluid to the microrollers could be the reason for such behavior^32^.
3. It was shown in the study that inertial forces arose for 25 and 50 μm, evidenced by Reynolds numbers (Fig. 3e,f), which could also be the source of non-linearities presented here.
4. The study approximated the repulsion forces as a combination of electrostatic repulsion force and van der Waals forces. However, it is important to note that other fundamental forces are present in the system, such as solvation forces, including hydrophobic/hydrophilic interactions or other steric effects, which were not taken into account. The origin of hydration or solvation forces differs from that of electrostatic or van der Waals forces, as they arise from the liquid medium and the physical and chemical properties of the interacting surfaces. The effect of these forces is less understood^27^. To accurately determine these forces, rigorous experimentation is required, and they cannot be modeled without determining experimental constants. Therefore, the presence of these forces could also contribute to the observed discrepancy, particularly at smaller lubrication distances.

Ideally, performing a dynamic-multiphysics simulation, including chemical and physical attraction forces and solid-state mechanics, could improve the results; nevertheless, it should be noted that it is exceptionally challenging to make a physical model capturing all features of the surface microrollers. Overall, the results presented here provided an improved understanding of microroller research from a practical perspective with their potential in drug delivery and lab-on-a-chip applications.

## Materials and methods

### Fabrication and actuation of surface microrollers

Magnetically actuated, spherical Janus microrollers were fabricated by sequentially sputtering Ni and Au nanofilms on pre-dried monolayer of silica (SiO_2_) particles of 4, 10, 25 and 50 μm diameter (Microparticles GmbH for 4, 10 and 25 μm, Corpuscular Inc.) using benchtop sputter coating system (Leica EM ACE600, Leica Microsystems). 1000 nm Ni was used for 4 and 10 μm, 1800 nm was Ni used for 25 and 50 μm particles, all microparticles had 50 nm Au as passivating layers. After sputtering, magnetization direction of the Janus microparticles was oriented towards out of the cap by applying a 1.8 T uniform magnetic field in a vibrating-sample magnetometer (VSM; MicroSense, Lowell, MA). Ni and Au nanofilm-sputtered microparticles were then released from the substrate via sonication in ethanol. The microrollers were intensely washed and finally dispersed in PBS 1 × . The microrollers were actuated using a custom-made five-coiled electromagnetic coil system placed on a microscope (Zeiss Axio Observer A1, Carl Zeiss). The microrollers were actuated using uniform rotating magnetic fields with an amplitude of 10 mT that enables surface rolling and steering control. All the experiments were performed in PBS 1 × with no confinements from any direction. The steady state translational speeds of the microrollers were analyzed using an in-house MATLAB tracking code.

### Computational fluid dynamics (CFD) analyses

COMSOL Multiphysics 6.0 Simulation Software (COMSOL, Inc.) was used to simulate microrollers and calculate the forces acting on the microrollers in all conditions, using laminar flow interface physics by solving the Navier–Stokes equations. We used previously validated simulations with increased resolution^2^. We defined two different mesh regions, the first one is around the microroller and the second one covered the remaining spaces in the simulation environment. 3*a* × 3*a* × 3*a*, where minimum element size, maximum element size, maximum element growth rate, curvature factor, and the resolution of narrow regions were defined in COMSOL as *a*/50, a/5, 1.2, 0.05, and 1.5 respectively for all sizes, where *a* is the radius of the microroller. For the second mesh, “extremely fine” built-in mesh settings were used in 40*a* × 40*a* × 40*a* dimensions. The density and the dynamic viscosity of the fluid were taken as $\rho$=1000 kg/m^3^ and *µ* = 1 cP. The boundaries other than microrollers’ were defined as no-slip boundaries. We either rotated or translated the spherical microrollers with diameters, 2*a,* of 5, 10, 25 and 50 µm to derivate the expressions for* F*_*P*_, *F*_*D*_ and *F*_*L*_. The hydrodynamic forces acting on the spheres were quantified in each case according to:

5 $$F=\iint{\varvec{\sigma}}\cdot {\varvec{n}} \mathrm{d}S$$

where $\sigma$ is the stress tensor, $n$ is the outward pointing normal vector, and *S* is the particle surface. The total acting force resulted in + *x* direction with clockwise rotation of the body, which is propulsion force, *F*_*P*_ and + *z* direction is the contribution of *F*_*L*_ from rotation^*2*^. The total acting force resulted in -*x* direction with the translation of the microrollers in + *x* direction is drag force, *F*_*D*_, and + *y* direction is the contribution of *F*_*L*_ from translation. The lift forces on the anisotropic shapes were calculated by taking the average of the forces over one rotation cycle.

## Supplementary Information

Supplementary Information.

## Data Availability

The datasets used and/or analysed during the current study available from the corresponding author on reasonable request.
