# Supplementary material for: The mismatch between experimental and computational fluid dynamics analyses for magnetic surface microrollers
Source: Sci Rep. 2023 Jun 23;13:10196. doi: 10.1038/s41598-023-37332-5 (PMC10290129; doi:10.1038/s41598-023-37332-5)
Supplement: Supplementary file 1 — Supplementary Information. [file 41598_2023_37332_MOESM1_ESM.docx]

**Supporting Information**

**for**

**The mismatch between experimental and computational fluid dynamics analyses for magnetic surface microrollers**

Ugur Bozuyuk^1,2^, Hakancan Ozturk^1^, Metin Sitti^1,2,3^*

^1^ Physical Intelligence Department, Max Planck Institute for Intelligent Systems, 70569 Stuttgart, Germany

^2^ Institute for Biomedical Engineering, ETH Zurich, Zurich 8092, Switzerland

^3^ School of Medicine and School of Engineering, Koç University, Istanbul 34450, Turkey

* Correspondence: [sitti@is.mpg.de](mailto:sitti@is.mpg.de)

**
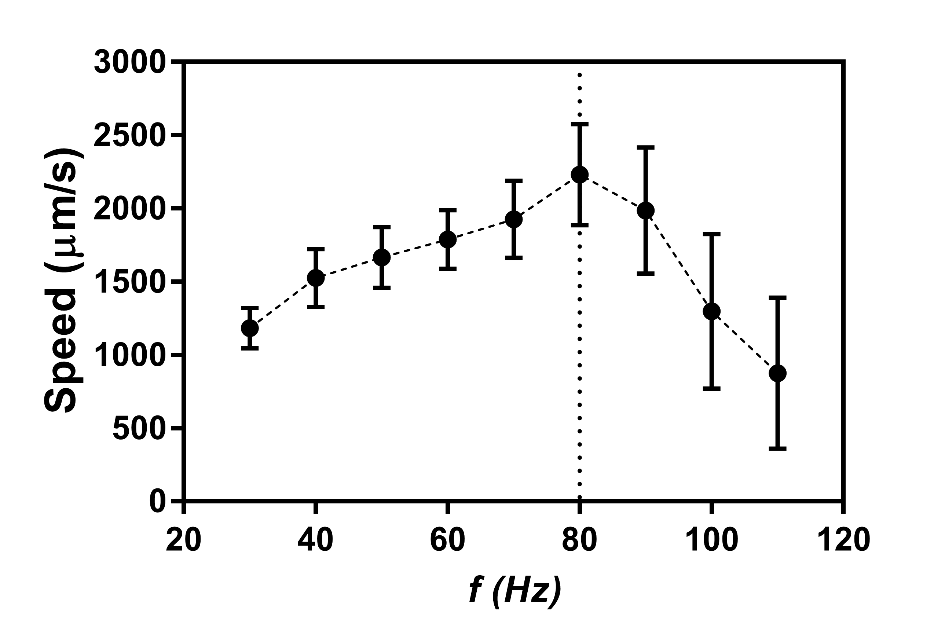
**

**Figure S1. Step out frequency for 50 μm microroller.** The step-out frequency for the 50 μm was determined as *f =*80 Hz, which was the highest actuation frequency used in the study.


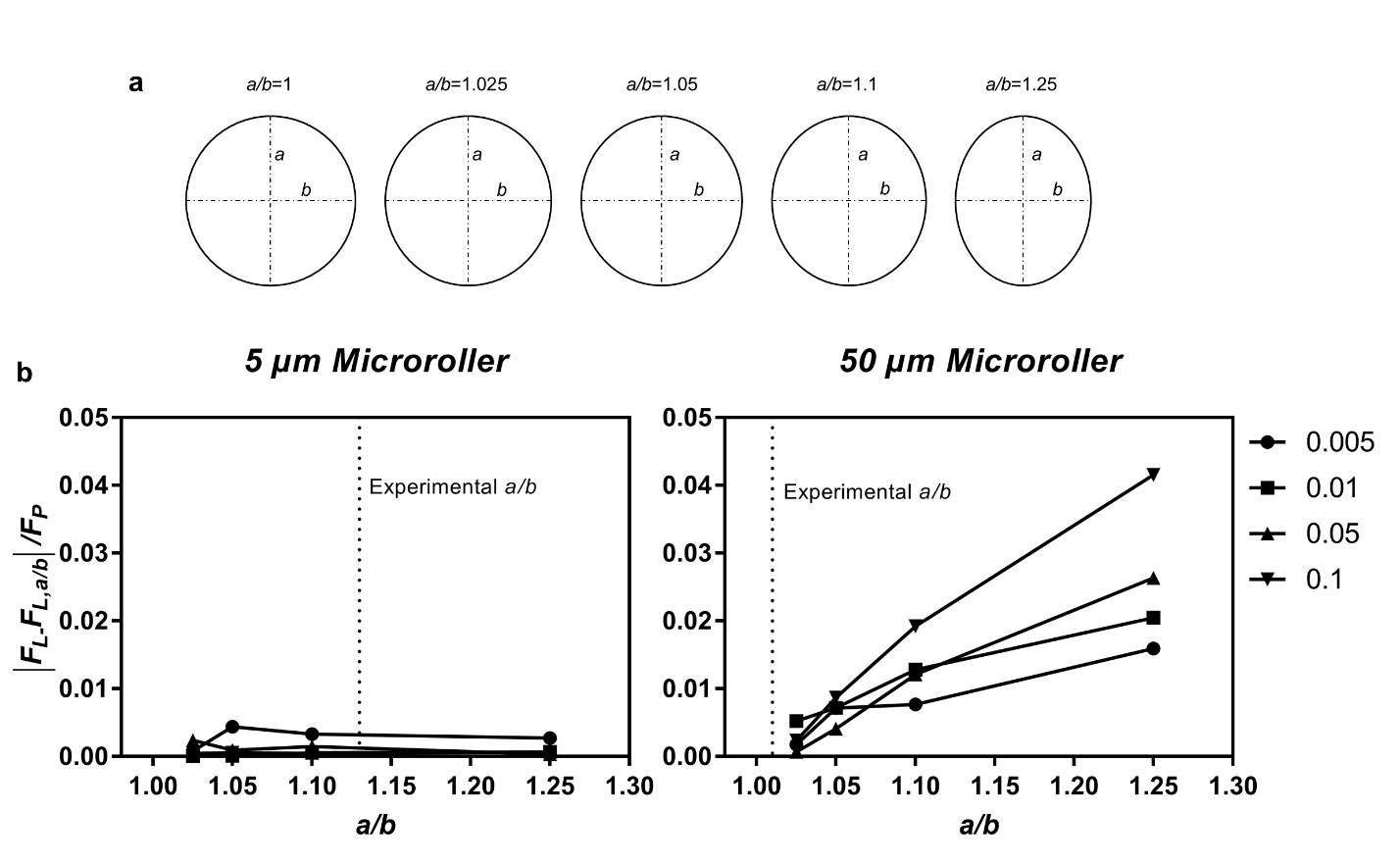


**Figure S2. CFD Simulations for anisotropic microrollers for their lift forces. a)** The anisotropic shapes used in the simulations. **b)** The lift force results for the shapes with different anisotropies. The lift forces calculated for anisotropic shapes (*F_L,a/b_*) were normalized and subtracted by lift force of the perfect sphere and normalized to the propulsion force of the perfect sphere. The lift force decreases were negligible for all anisotropies and followed an insensitive pattern due to the absence of inertia for 5 μm microroller. The lift force decreased with increasing anisotropy for 50 μm microroller. The experimental *a/b* values were marked in the graphs for both microroller sizes, based on Figure 2b.

**Supplementary Note 1**

The electrostatic repulsion force exerted on a microroller is expressed by: ^1^

$F_{es}=4\pi\varepsilon\varepsilon_{0}a\kappa v_{1}v_{2}e^{-\kappa\delta}$ *(S. Eq. 1)*

where $\varepsilon$ is the permittivity of the medium, $\varepsilon_{0}$ is the vacuum permittivity, *a* is radius of the microroller, $\kappa^{-1}$is Debye length, $v_{1}$ and $v_{2}$ are the zeta potential of the particles and the near-surface, respectively*,* $\rho_{f}$ is the density of the fluid*,* $\rho_{p}$, density of the particle and *g* is the gravitational constant. We performed all the experiments in PBS 1×. The corresponding values are $\varepsilon$=80, $\varepsilon_{0}$=8.854 x 10^-12^ F/m, $\kappa^{-1}$ = 0.7 nm. The zeta potentials were assumed as $v_{1}$=-7.37 mV, $v_{1}$=-70 mV based on the previous works. ^1-2^

van der Waals forceon a spherical object near a flat surface is expressed by^4^:

$F_{vdW}=-Aa/6\delta^{2}$ *(S. Eq. 2)*

Where *A* is Hamaker constant, a is radius of the microroller, and $\delta$ is the lubrication distance. The Hamaker constant for our system was approximated to be *A*=7×10^-23^ J, based on previous work for micron-sized silica particles^5^.

**Supplementary Note 2**

We modeled anisotropic microrollers with different anisotropy ratios, *a/b*=1, 1.025, 1.05, 1.1 and 1.25 (**Figure S2a**). and 1.25 for 5 and 50 μm microrollers, while they have *a/b*=1.13 and 1.01 respectively (**Figure 2b**). The translational speed of the microrollers was mainly determined by lubrication distance, rotation frequency and diameter. Lubrication distance is changed by the forces on the *z-*axis. The lift forces acting on the anisotropic microrollers would be different, and that could contribute to the changes in lubrication distances and, thus discrepancies. The simulations for the lift force quantification for different anisotropy ratios have shown that the lift was negligible for the 5 μm mainly due to the absence of inertia, and the changes were not sensitive to the changes in anisotropy ratio (**Figure S2b**), as expected in the such regime for the forces in *z-*direction^6^. On the other hand, the lift force decreases with increasing anisotropy for 50 μm; however, the 50 μm microroller had a very small anisotropy ratio; it was almost a perfect sphere with *a/b*=1.01 (**Figure 2b**). Thus, the small anisotropies could contribute to such discrepancies; however, the results for 5 μm did show significant lift force differences.

**References**

1. Chiang, T.-Y.; Velegol, D., Localized Electroosmosis (LEO) Induced by Spherical Colloidal Motors. *Langmuir* **2014,** *30* (10), 2600-2607.

2. Alapan, Y.; Bozuyuk, U.; Erkoc, P.; Karacakol, A. C.; Sitti, M., Multifunctional surface microrollers for targeted cargo delivery in physiological blood flow. *Science Robotics* **2020,** *5* (42), eaba5726.

3. Dou, Y.; Tzelios, P. M.; Livitz, D.; Bishop, K. J. M., Programmable topotaxis of magnetic rollers in time-varying fields. *Soft Matter* **2021,** *17* (6), 1538-1547.

4. Israelachvili, J. N. Intermolecular and surface forces. (Academic press, 2011).

5. Valmacco, V., Elzbieciak-Wodka, M., Besnard, C., Maroni, P., Trefalt, G., & Borkovec, M. (2016). Dispersion forces acting between silica particles across water: Influence of nanoscale roughness. *Nanoscale Horizons* **2016**, *1*(4), 325-330.

6. Fang, W. Z., Ham, S., Qiao, R., & Tao, W. Q. (2020). Magnetic actuation of surface walkers: The effects of confinement and inertia. *Langmuir* **2020**, 36(25), 7046-7055.
